# Introducing Medical Students into the Emergency Department: The Impact upon Patient Satisfaction

**DOI:** 10.5811/westjem.2015.9.27255

**Published:** 2015-11-22

**Authors:** Christopher Kiefer, Joseph S. Turner, Shelley M. Layman, Stephen M. Davis, Bart R. Besinger, Aloysius Humbert

**Affiliations:** * West Virginia University School of Medicine, Department of Emergency Medicine, Morgantown, West Virginia; † Indiana University School of Medicine, Department of Emergency Medicine, Indianapolis, Indiana

## Abstract

**Introduction:**

Performance on patient satisfaction surveys is becoming increasingly important for practicing emergency physicians and the introduction of learners into a new clinical environment may impact such scores. This study aimed to quantify the impact of introducing fourth-year medical students on patient satisfaction in two university-affiliated community emergency departments (EDs).

**Methods:**

Two community-based EDs in the Indiana University Health (IUH) system began hosting medical students in March 2011 and October 2013, respectively. We analyzed responses from patient satisfaction surveys at each site for seven months before and after the introduction of students. Two components of the survey, “Would you recommend this ED to your friends and family?” and “How would you rate this facility overall?” were selected for analysis, as they represent the primary questions reviewed by the Center for Medicare Services (CMS) as part of value-based purchasing. We evaluated the percentage of positive responses for adult, pediatric, and all patients combined.

**Results:**

Analysis did not reveal a statistically significant difference in the percentage of positive response for the “would you recommend” question at both clinical sites with regards to the adult and pediatric subgroups, as well as the all-patient group. At one of the sites, there was significant improvement in the percentage of positive response to the “overall rating” question following the introduction of medical students when all patients were analyzed (60.3% to 68.2%, p=0.038). However, there was no statistically significant difference in the “overall rating” when the pediatric or adult subgroups were analyzed at this site and no significant difference was observed in any group at the second site.

**Conclusion:**

The introduction of medical students in two community-based EDs is not associated with a statistically significant difference in overall patient satisfaction, but was associated with a significant positive effect on the overall rating of the ED at one of the two clinical sites studied. Further study is needed to evaluate the effect of medical student learners upon patient satisfaction in settings outside of a single health system.

## INTRODUCTION

In 2006, in response to the growing shortage of physicians in the United States, the Association of American Medical Colleges advocated for a 30% increase in enrollment at Liaison Committee on Medical Education (LCME) accredited institutions by the year 2015.^1^ As of 2012 LCME schools were on target to reach the goal.^2^

In addition to increased enrollments, demand for emergency medicine (EM) training venues is also created by the increasing number of required EM clerkships at LCME schools, having risen from 33% to 52% of schools between 2003 and 2010.^3^,^4^ Since most medical students currently complete their EM clerkship in large, academic, tertiary care hospitals where resident physicians are also present, institutions may need to increasingly utilize alternative clinical settings, including community-based emergency departments (EDs) as training sites for medical students.

As community EDs begin to host an increasing number of medical student rotators, concerns have arisen regarding the impact upon patient experience and satisfaction. Prior work has illustrated that although community-based EDs may have lower patient volumes than the primary university site, they are viable teaching sites, as students rotating at community-based sites have a significantly higher number of patients evaluated per shift, a significantly higher number of procedures per shift, and gave higher clinical teaching scores to attending physicians.^5^

Attending physicians practicing in community-based EDs are facing increased pressure to perform well on patient satisfaction surveys. The introduction of value-based purchasing (VBP) by the Center for Medicare Services (CMS) has tied portions of hospital reimbursement to patient satisfaction. The results of patient satisfaction surveys may be used by hospitals to make credentialing decisions regarding individual physicians, make bonus payments to physicians, as well as to determine the contract status for an entire group of physicians.^6^ It has been hypothesized that attending physicians in this setting may be hesitant to participate in teaching experiences, fearing the negative impact that the presence of learners will have upon patient satisfaction.^7^

While it appears that community EDs are viable venues for clinical education, physicians in this setting are also interested in maintaining high patient satisfaction. To our knowledge, there has been no prior work that evaluates the relationship between patient satisfaction and the introduction of medical students to the community ED setting. This study seeks to examine the effect of introducing medical students into two community-based EDs upon performance on institutional patient satisfaction surveys.

## METHODS

At Indiana University, EM is a required clerkship for all fourth-year medical students that may be completed at a variety of clinical sites – ranging from large, tertiary academic centers to smaller community-based ED settings throughout Indiana. Two community-based EDs in the Indiana University Health (IUH) system, IUH North (Site A) and IUH Saxony (Site B), were chosen for analysis in this study given that prior to the start date of medical students in the ED, there were no medical students or residents present in the hospital. During the study period, both sites were staffed using a single coverage model, where there was a single American Board of Emergency Medicine-certified physician working at all times. While there was not a medical student present at either site during all shifts, all physicians at both sites worked with medical students when a student was present. The study was designed as a retrospective cohort of existing patient satisfaction data collected by both sites during the study period as part of standard ED operations.

Patient satisfaction surveys were administered to patients discharged from the EDs in the study by a third party, National Research Corporation (NRC) Picker. The surveys were administered by NRC Picker to discharged patients from both ED sites via US mail as per the pre-existing contract between the institution and NRC Picker. No additional patient satisfaction surveys were administered during the study period. Surveys were returned to NRC Picker by individual patients at their discretion. If an individual patient did not answer a question on the survey, we excluded that data point from analysis for that particular question.

Survey results were reported to the clinical sites on a monthly basis during the study period as a percentage of positive response to each question. The standard report provided to both clinical sites reported the percentages of positive response for adult, pediatric, and all patients.

We analyzed surveys for the seven months before and after the introduction of medical students at each clinical site with the study powered to detect a 15% difference in positive response rate. Medical students were introduced at Site A in March 2011 and at Site B in October 2013. As such, studies collected from August 2010–February 2011 and March 2011–October 2011 formed the pre- and post-medical student cohorts at Site A with surveys from February 2013–August 2013 and October 2013–April 2014 forming the pre- and post-medical student cohorts at Site B.

For purposes of the study, two questions on the survey were subjected to data analysis–“Would you recommend this ED to your friends and family?” and “How would you rate this facility overall?”–where a positive response was considered a 9 or 10 on a scale of 1–10 or a score of excellent prior to January 2011 (Site A only). These questions were chosen for analysis as they represent the primary outcome measures reported by the institution as part of CMS VBP and the results to these questions are publicly reported. “Definitely yes” was considered a positive response for the “would you recommend” question. Responses of 9, 10, or excellent (prior to January 2011) considered as a positive response for the “how would you rate” question. The change from a categorical response “excellent/definitely yes” to a continuous response “9 or 10” represented a change in the survey response options implemented by the survey vendor. As the survey results before and after this alteration were reported as a percentage of positive response, the change in terminology does not represent a change in the primary outcome, and as such, we included both survey sets in the analysis.

We compared percentages of positive response for the periods before and after the introduction of medical students using a chi-square analysis. This study was deemed exempt by the institutional review board at the Indiana University School of Medicine.

## RESULTS

For Site A, 224 surveys were returned in the seven months prior to the introduction of medical students and 520 surveys returned in the seven months following the introduction of medical students. For Site B, there were 247 surveys returned prior to the introduction of the students with 224 surveys returned following the introduction of medical students. The survey response rate was 22.8% for Site A for January 2011–October 2011and 17.2% for Site B for the entirety of the study period. Response rate data prior to January 1, 2011 for Site A was not available. The surveys in this study were administered by a third party; only the total number of surveys returned and the rate of return were reported to the institutions. As such, we were unable to directly ascertain the reasons behind the increased total number of surveys at Site A in the post-medical student period.

Two individuals at Site A and three individuals at Site B did not answer the “would you recommend” question in the pre-student period. Following the introduction of students, two individuals at Site A and six individuals at Site B did not complete the “overall rating” component of the survey.

For Site A, we were unable to detect a statistically significant difference in the percentage of positive response for the “would you recommend” question when the adult (p=0.549), pediatric (p=0.284), or all-patient (p=0.238) groups were analyzed (Table 1). For the same query at Site B, we were again unable to detect a statistically significant difference in the percentages of patients giving a positive response to the “would you recommend” question in the adult (p=0.353), pediatric (p=0.758), or all patients (p=0.756) (Table 2).

Regarding the “overall rating” component of the survey, when we analyzed all patients at Site A, the percentage of patients giving an overall rating of “excellent” or “9/10 and 10/10” increased from 60.3 to 68.2 (p=0.038). However, when broken down into subgroups, we were unable to detect a statistically significant difference between the adult (p=0.347) or pediatric (p=0.062) groups (Table 1). For the same measure at Site B, we were unable to detect a statistically significant difference in the adult (p=0.738), pediatric (p=0.554), or all patient (p=0.476) groups (Table 2).

Table 3 illustrates the pooled patient satisfaction data from both sites. For the “would recommend” question, we were unable to detect a statistically significant difference in the percentage of positive response in the adult (p=0.976), pediatric (p=0.203), or all patient groups (p=0.333). Finally, when analyzing the pooled data for the “overall rating” question, we were unable to detect a statistically significant difference in the before or after medical student groups in the adult (p=0.817), pediatric (p=0.791), or all patient (p=0.625) groups.

## DISCUSSION

Patient satisfaction is becoming increasingly important for the practicing emergency physician. To date, no prior study has analyzed the effect of medical student learners on patient satisfaction in the ED setting. In both EDs, there were no medical students or residents present anywhere in the hospital prior to the start date of medical students. While most teaching settings do not have a discrete start date for learners in their facility, the lack of students prior to a certain time point allows for a direct evaluation of the impact of medical students upon patient satisfaction scores.

The addition of learners to a clinical environment adds an additional step in the patient’s visit to the ED where the student first evaluates the patient and subsequently presents the findings to an attending physician. Previous work has illustrated that while medical students reduce the time to medical provider, the total length of stay for patients seen by a medical student in the ED is increased by an average of 24 minutes.^8^ Total length of stay is certainly an important metric that many EDs follow closely; however, reduced door-to-provider time has been associated with increased patient satisfaction scores.^9^ Our study did not measure door-to-provider time directly; however, in a single coverage ED, it is possible that medical students impact this positively and may be an area of additional study.

In the past, patients have generally viewed the involvement of medical students in their care positively. Prislin et al. evaluated patient perceptions of medical student participation in a family medicine clerkship at both community-based and tertiary academic clinics–89% of surveyed patients responded that being seen by a medical student was an enjoyable experience and 77% of patients responded that they felt medical student participation improved the quality of care they received.^10^ More recently, a Colombian group found that the introduction of medical students into the inpatient setting improved patient perception of quality of care and overall satisfaction.^11^ While our study failed to detect a significant positive difference in most of our measured outcomes, the lack of a negative effect may be reassuring for community-based physicians considering becoming involved in medical student education. Given the increasing demand for medical student education sites and increasing viability of community-based EDs as clinical teaching sites, our results suggest that the presence of medical students does not affect patient satisfaction scores, and patient satisfaction alone should not be considered a barrier to introducing medical students into clinical venues.

## LIMITATIONS

Our study has several limitations. First, this study was performed at two sites within the same health system and used only NRC Picker survey results, and the results may not be generalizable. The analyzed outcomes assessed patient satisfaction with their entire visit. We believe that this is an appropriate measure of patient satisfaction as they represent the primary outcome measures reported by the institution as part of CMS VBP and therefore are meaningful outcomes to the institution and practicing physicians. However, multiple possible confounders could affect the patient’s impression of the entire visit. We also did not evaluate individual physician satisfaction scores, though this is an area for potential, future study. This study analyzed a relatively short time period, and does not evaluate larger trends in satisfaction scores. We acknowledge that many health systems are applying service initiatives to increase scores, and this “snapshot” does not evaluate the effect of such efforts.

Due to the fact that patient satisfaction surveys are de-identified, we were unable to focus our analysis on only those patients evaluated by a medical student. Although creation of a separate survey instrument with similar queries would allow for analysis of patients evaluated by a medical student, our study is, to our knowledge, the first to analyze the impact of introducing learners into a clinical environment upon data used for CMS reporting and quality metrics. Finally, our study was powered to detect a 15% difference. While this is a sizable difference, it was chosen in part because Site B is a newly opened hospital, with a limited time of pre-learner data. This limits our ability to detect small differences over a long period of observation.

## CONCLUSION

This study suggests that introducing medical students into a community ED does not have a significant impact on patient satisfaction scores. With increasing emphasis on patient satisfaction, the results of this study suggest that sites considering participating in medical student training should be assured that students do not have a negative impact upon patient satisfaction.

## Figures and Tables

**Table 1:** Percentage of positive responses to the “would you recommend” and “Overall rating” items at Site A before and after the introduction of medical students.

	“Would you recommend?”		“Overall rating”	
	Before students	After students	Before students	After students
Pediatrics	84.1 (n=113)	79.3 (n=256)	50.9 (n=114)	61.3 (n=253)
Adult	84.4 (n=109)	81.9 (n=264)	70.0 (n=110)	74.7 (n=265)
Overall	84.2 (n=222)	80.6 (n=520)	60.3 (n=224)	68.2 (n=518)*

* Differences in percentages were found to be statistically significant (p<0.05).

**Table 2:** Percentage of positive responses to the “would you recommend” and “Overall rating” items at Site B before and after the introduction of medical students.

	“Would you recommend?”		“Overall rating”	
	Before students	After students	Before students	After students
Pediatrics	84.85 (n=99)	83.1 (n=71)	81.82 (n=99)	85.29 (n=68)
Adult	83.11 (n=148)	86.93 (n=153)	83.45 (n=145)	84.87 (n=152)
Overall	83.98 (n=247)	85.02 (n=224)	82.64 (n=244)	85.08 (n=220)

**Table 3:** Combined percentages of positive responses for both clinical sites before and after the introduction of medical students.

	“Would you recommend?”		“Overall rating”	
	Before students	After students	Before students	After students
Pediatrics	84.85 (n=212)	80.13 (n=327)	65.27 (n=213)	66.38 (n=321)
Adult	83.66 (n=257)	83.75 (n=417)	77.65 (n=255)	78.41 (n=417)
Overall	84.08 (n=469)	81.93 (n=744)	71.95 (n=468)	73.23 (n=738)
